# Upper body motor function and swallowing impairments and its association in survivors of head and neck cancer: A cross-sectional study

**DOI:** 10.1371/journal.pone.0234467

**Published:** 2020-06-19

**Authors:** Lucía Ortiz-Comino, Carolina Fernández-Lao, Caroline M. Speksnijder, Mario Lozano-Lozano, Isabel Tovar-Martín, Manuel Arroyo-Morales, Lydia Martín-Martín

**Affiliations:** 1 Department of Physical Therapy, University of Granada, Granada, Spain; 2 Department of Physical Therapy, Instituto Biosanitario Granada (IBS Granada), Instituto Mixto Deporte y Salud (iMUDS), University of Granada, Granada, Spain; 3 Department of Head and Neck Surgical Oncology, University Medical Center Utrecht, Cancer Center, Utrecht University, Utrecht, The Netherlands; 4 Department of Oral and Maxillofacial Surgery and Special Dental Care, University Medical Center Utrecht, Utrecht University, Utrecht, The Netherlands; 5 University Medical Center Utrecht, Julius Center for Health Sciences and Primary Care, Utrecht University, Utrecht, The Netherlands; 6 Department of Physical Therapy, Instituto Biosanitario Granada (IBS Granada), University of Granada, Granada, Spain; 7 Radiation Oncology Department, Virgen de las Nieves University Hospital, Granada, Spain; Universiteit Antwerpen, BELGIUM

## Abstract

**Background:**

Upper body motor function and swallowing may be affected after curative treatment for head and neck cancer. The aims of this study are to compare maximum mouth opening (MMO), temporomandibular dysfunction (TMD), cervical and shoulder active range of motion (AROM) and strength, and swallowing difficulty between survivors of head and neck cancer (sHNC) and healthy matched controls (HMC) and to examine the correlations between these outcomes in sHNC.

**Methods:**

Thirty-two sHNC and 32 HMC participated on the study. MMO, TMD, cervical and shoulder AROM, cervical and shoulder strength, the SPADI shoulder pain and disability indices, the Eating Assessment Tool (EAT-10) score, swallowing difficulty as determined using a visual analogue scale (VAS), and the location of disturbances in swallowing, were recorded.

**Results:**

MMO and cervical and shoulder AROM and strength were significantly lower in sHNC, whereas FAI, SPADI score, EAT-10 and VAS were higher. The MMO, TMD, cervical and shoulder AROM, and cervical shoulder strength values showed significant correlations (some direct, others inverse) with one another. Swallowing difficulty was inversely associated with the MMO, cervical AROM and shoulder strength.

**Conclusion:**

Compared with controls, sHNC present smaller MMO, lower cervical and shoulder AROM, lower cervical and shoulder strength and higher perception of TMD, shoulder pain and disability and swallowing difficulty. sHNC suffer impaired swallowing related to lower MMO, presence of TMD, cervical AROM and shoulder strength values. Improving these variables via physiotherapy may reduce the difficulty in swallowing experienced by some sHNC.

## Introduction

Head and neck cancer (HNC) refers to cancer of the nasal cavity and paranasal sinuses, the oral cavity, the salivary glands, pharynx and larynx. The worldwide incidence of HNC is some 650,000 cases per year; in Europe, this incidence reaches 140,000 cases, whereas in Spain HNC incidence is around 10,000 cases annually [1]. It is more common in men than in women; the mean age at diagnosis is 50 years [2]. The most common risk factors for HNC are tobacco use, alcohol consumption and infection with human papillomavirus [3,4]. Treatment for HNC commonly involves surgery, radiotherapy (RT), chemotherapy, or a combination of these. Intraoperative procedures causing stretching, compression or burning (from electrocauterization) of soft and neural tissues can lead to neuropraxia or axonal injury, as can post-operative scarring, haemorrhages and infections [5] potentially impairing motor function in the affected region and surrounding areas with an impact on oral and oropharyngeal functioning [5]. When lymph node metastases are suspected, a neck dissection (ND) may be performed, either selective ND (SND), modified radical (MRND), or radical ND (RND) depending on requirements, in addition to the resection of the primary tumor surgery [6]. SND, which involves cervical lymphadenectomy, preserves one or more of the lymph node groups that are usually removed in RND. In MRND, all the local lymph nodes are removed, but one or more non-lymphatic structures are preserved (e.g., the spinal accessory nerve, the internal jugular vein or the sternocleidomastoid muscle). RND involves the extirpation of all ipsilateral lymph node groups from the lower border of the mandible to the clavicle, as well as the removal of the spinal accessory nerve, the internal jugular vein and the sternocleidomastoid muscle [7].

Radiotherapy may induce the formation of collagen, leading to a thickening of the dermis and, therefore, fibrosis [8], which may cause a loss of function of the masticatory system [9]. Muscle contractures may also appear as a consequence of radiation-induced fibrosis in the neck and shoulder regions, which reduces their motor functioning.

Adequate skeletal support and muscle function are essential to swallowing [10]. If the oral cavity and pharyngeal muscles are damaged by surgery or radiotherapy, dysphagia, may result [11]. The toxic effects of chemotherapy—which is commonly prescribed in the treatment of locally advanced disease may also hinder patient recovery and swallowing ability [12].

Swallowing impairments related to the presence of trismus (maximum mouth opening [MMO] ≤35 mm [13]) have been studied in survivors of head and neck cancer (sHNC) [14], but it is unknown whether any associations exist between MMO, temporomandibular dysfunction (TMD), cervical and shoulder motor function, and swallowing impairments in sHNC.

Therefore, the primary aim of this case-control study is to test if there are differences between sHNC and healthy age- and sex-matched controls for MMO, TMD, cervical and shoulder motor function, and swallowing impairments. The secondary aim is to analyse in sHNC the association between MMO, TMD, cervical and shoulder motor function, and swallowing impairments.

## Methods

### Study subjects

The sHNC were recruited at the Virgen de las Nieves University Hospital, Granada (Spain). All met the following inclusion criteria: 1) age ≥18 years, 2) curative treatment completed in the previous 6–36 months, 3) shoulder and/or cervical dysfunction present, and 4) a tumor located (before treatment) in the nasal cavity, either paranasal sinus, nasopharynx, oral cavity, oropharynx, hypopharynx, or larynx. The exclusion criteria were: 1) having a metastasis or active neoplasm, 2) previous neck and/or shoulder impairments, and 3) cognitive impairment.

The control group was formed by healthy age-and sex matched volunteers who responded to announcements for the study. They were excluded if they reported a history of cervical, shoulder and/or TMJ pain, a history of trauma, or if they had any systemic disease. The present study was approved by the Biomedical Investigation Ethics Committee, Granada, Spain (CEi-GRANADA Ref: 0045-N-16) and conducted in accordance with the Declaration of Helsinki. All patients gave written informed consent before being formally enrolled.

### Subject demographic and clinical data

Demographic (age, sex, tobacco and alcohol consumption) and clinical data (cancer location and tumor stage at diagnosis [15], time since diagnosis, affected side, kind of curative cancer treatment) were recorded at the appointment with the patient. Affected side was described as ipsilateral side and unaffected side as contralateral side [16].

Data on smoking habit (non-smoker, smoker, or ex-smoker) and alcohol consumption (none, monthly, weekly and daily) were also recorded.

### Assessment

During the assessment MMO, cervical active range of motion (AROM) and muscle strength, and shoulder AROM and muscle strength were measured by objective tests. TMD, shoulder pain and disability, and swallowing impairments were measured by questionnaires. Also a visual analogue scale (VAS) was used to measure the experience of swallowing difficulty.

### Maximum mouth opening

MMO was measured (mm) once as the inter-incisor distance (with the patient sitting) using a sliding caliper [17,18].

### Temporomandibular dysfunction

TMD was assessed using the Fonseca Anamnestic Index (FAI), a commonly employed screening tool for TMD [19]. It consists of 10 self-administered questions with three possible responses: yes, sometimes or no. A total score of 0 reflects no TMD, 1 indicates mild dysfunction, 2 moderate dysfunction, and 3 severe dysfunction.

### Cervical function

The cervical active range of motion (AROM) was measured by examining cervical flexion, extension, inclination and rotation (towards both sides) using a cervical range of motion device (Performance Attainment Associates©, Spine Products, Roseville, MN, USA). During testing patients sat in an upright position.

Cervical muscle strength was determined using the deep cervical flexor endurance test (DCFET). Briefly, subjects started in a supine position with the examiner’s hands under their head. They then performed an upper-cervical extension (i.e., making a double chin), raising the head as little as possible from the examiner’s hands. The time elapsed from when the patient raised the head until 1) the adopted posture could no longer be maintained, 2) the patient’s head rested on the examiner’s hands for more than 1 s, or 3) the patient started to feel pain, was recorded [20]. This test has an intraclass coefficient (ICC) of 0.82–0.91 [21].

### Shoulder function

Shoulder AROM was measured (both sides) using a two-arm goniometer with a 360° protractor [22] examining shoulder flexion, abduction, external and internal rotation with the patient lying in the supine position. In each test, patients were instructed to move the joint from a neutral position to the end of their range (i.e., until pain or stiffness appeared), avoiding compensation movements. Each movement was examined once and the angle reached recorded [23]. The ICC of the goniometer used is excellent (0.94) [24].

Shoulder muscle (upper trapezius) strength was measured using the Daniels and Worthingham’s muscle test scale [25], scoring from absence of contraction (0) to normal muscle response (5). Tests were again performed bilaterally. Briefly, patients sat in an upright position with both arms resting by the trunk, and pushed as strongly as possible with their shoulder towards the ceiling, against the examiner’s hand (with the score determined by the examiner) This test has an ICC of 0.63–0.98 [26].

The shoulder pain and disability index (SPADI) was determined for all subjects. The SPADI is a 13-item, self-administered questionnaire with two subscales: pain and disability. Subjects report the pain and disability experienced in the previous week. Each item is rated on a 0–10 scale, from no pain/no dysfunction (0) to maximum pain/impossible (10). The scores for pain and disability are calculated from the sum of the corresponding items divided by the maximum score possible and multiplied by 100. To obtain the total score, the mean of the pain and disability scores is calculated. The SPADI has been validated for use in populations with shoulder pain and has an ICC of ≥0.89 [27]. The SPADI Spanish version [28] has been validated for general use, and has been used for populations with HNC [18,29].

### Swallowing impairments

The Eating Assessment Tool (EAT-10) was used to evaluate self-reported swallowing impairments. This tool rates 10 items on a 5-point Likert scale from 0 (no impairment) to 4 (severe problem). The sum of the scores for these 10 items provides the overall score; impaired swallowing is reflected by a score of ≥3 [30]. The ICC for this test is 0.72–0.91 for a wide range of populations with swallowing disorders, including patients with HNC [30].

Swallowing difficulty was also assessed using a 10 cm-long visual analogue scale (VAS) (0 = no difficulty, 10 = impossible to swallow) (10). Patients were also asked where they felt the problem existed when swallowing (pre-oral [i.e., mouth opening], oral, pharyngeal, or all three places).

### Statistical analysis

Continuous data were expressed as means and standard deviations, ordinal and categorical data as numbers and percentages. The distribution of all variables was determined using the Kolmogorov-Smirnov test. Differences between sHNC and healthy age- and sex-matched controls in continuous and normal distributed data were analyzed with the independent T-test. The Mann-Whitney U test was used for ordinal and non-normal distributed continuous data. Differences on categorical variables between sHNC and healthy controls were analyzed by the Chi^2^ test.

Pearson’s correlation coefficient was determined to identify associations between normally distributed variables; Spearman’s correlation coefficient was determined when any variable in a pair was not normally distributed. Correlation analysis for cervical and shoulder AROM variables were performed using the mean values for the left and right sides. Significance was set at p<0.05. All calculations were performed using SPSS 25.0 software (IBM Corp., Armonk, NY, USA).

All p-values lower than 0.05 were considered statistically significant. Tests were performed with software SPSS 25.0 (Chicago, Illinois).

## Results

### Demographic and clinical data

Thirty-two sHNC, 12 women and 20 men, and 32 healthy age- and sex-matched controls were recruited; their mean age was 58.8±11.9 for the sHNC group and 58.4±12 for the control group. Eleven HNC and 19 controls did not smoke, 4 sHNC and 4 controls were smoker at the assessment time, and 17 sHNC were ex-smoker compared to 9 ex-smoker controls. No statistically significant differences were found for the smoking habits, but we did find statistically significant difference over alcohol consumption between groups (*p* = 0.004), with the sHNC group drinking less often than the control group. Twenty eight sHNC received surgery, 11 of whom also received radiotherapy, and 16 of whom also received chemoradiotherapy (CRT). Four received CRT only. Twenty two sHNC underwent ND. The mean time between diagnosis and assessment was 21.1±10.7 months. Tables 1 and 2 summarize the subjects' demographic and clinical characteristics.

**Table 1:** Subjects’ demographic data. Continuous data are expressed as means (SD), and categorical data as numbers (%). sHNC: survivors of head and neck cancer.

	sHNC (N = 32)	Healthy matched controls (N = 32)	*p*-value
Age (years)	58.8 (11.9)	58.4 (12)	0.977^‡^
Gender			0.602^μ^
Female	12 (37.5)	12 (37.5)	
Male	20 (62.5)	20 (62.5)	
Smoking habits			0.101^μ^
Non-smoker	11 (34.4)	19 (59,4)	
Smoker	4 (12.5)	4 (12.5)	
Ex-smoker	17 (53.1)	9 (28.1)	
**Alcohol consumption**			**0.004**^μ^
No consumption	15 (46.9)	10 (31.3)	
Monthly	8 (25)	3 (9.4)	
Weekly	3 (9,4)	16 (50)	
Daily	6 (18.8)	3 (9.4)	

^‡^: independent t-test

^μ^: Chi^2^ test

**Table 2:** sHNC clinical data. Continuous data are expressed as means (SD), and categorical data as numbers (%). CRT: Chemoradiotherapy; MRND: modified radical neck dissection; RND: radical neck dissection; RT: radiotherapy; sHNC: survivors of head and neck cancer.

	sHNC
	(N = 32)
Tumor location	
Nasal cavity, paranasal sinuses and nasopharynx	2 (6.2)
Oral cavity and oropharynx	
Larynx and hypopharynx	19 (59.4)
	11 (34.4)
Tumor stage	
I	6 (18.8)
II	6 (18.8)
III	7 (21.9)
IV	13 (40.5)
Time since diagnosis (months)	21.1 (10.7)
Treatment modality	
RT	1 (3.1)
CRT	3 (9.4)
Surgery	1 (3.1)
Surgery + RT	11 (34.4)
Surgery + CRT	16 (50)
Type of neck dissection	
None	10 (31.3)
MRND	15 (46.9)
RND	7 (21.8)

### Maximum mouth opening

MMO was statistically significant different between sHNC and healthy-matched controls (*p* = 0.002). On average sHNC reached 34.5mm (±13.3) which is scored as the presence of trismus, whereas the control group reached 44.1mm (±7.4). AMMO for 17 sHNC and 5 controls was scored equal or below 35mm, therefore this was scored as trismus presence. Fifteen sHNC and 27 controls scored the absence of trismus with a MMO at least 35mm (Table 3).

**Table 3:** MMO, TMD, cervical, shoulder and swallowing function results. Continuous data are expressed as means (SD), and categorical data as numbers (%). AROM: active range of motion; DCFET: deep cervical flexor endurance test; EAT-10: Eating Assessment Tool; FAI: Fonseca Anamnestic Index; MMO: maximum mouth opening; sHNC: survivors of head and neck cancer; SPADI: shoulder pain and disability index; VAS: visual analogue scale.

	sHNC (N = 32)	Healthy matched controls (N = 32)	*p*-value
**MMO**	34.5 (13.3)	44.1 (7.4)	**0.002**^**‡**^
**FAI**			**0.001**^**μ**^
No dysfunction	12 (37%)	24 (75%)	
Light dysfunction	7 (22%)	8 (25%)	
Moderate dysfunction	6 (19%)	0 (0%)	
Severe dysfunction	7 (22%)	0 (0%)	
Cervical AROM			
Flexion	42.4 (11.7)	45.7 (16.9)	0.561^**‡**^
**Extension**	46.8 (14.4)	57.8 (15.6)	**0.007**^**‡**^
**Inclination to affected side**	31.9 (11.6)	39.1 (9.9)	**0.014**^**‡**^
**Inclination to unaffected side**	31.6 (8.5)	39.1 (9.9)	**0.003**^**‡**^
Rotation to affected side	49.8 (14.8)	58.4 (15.9)	0.077^†^
Rotation to unaffected side	52.5 (12.7)	55.9 (15.7)	0.194^†^
**DCFET**	10.5 (6.8)	27.8 (14.6)	**0.001**^**‡**^
Shoulder AROM			
**Flexion affected**	149.5 (29.9)	163.5 (16)	**0.016**^†^
Flexion unaffected	155.1 (23.9)	165.9 (13.6)	0.172^†^
**Abduction affected**	138.3 (36.8)	159.4 (23.3)	**0.014**^†^
Abduction unaffected	145.6 (32.4)	164.7 (20.9)	0.130^†^
External rotation affected	74.2 (17.9)	80 (16.5)	0.198^†^
External rotation unaffected	72.8 (20.4)	80.9 (16.2)	0.135^†^
Internal rotation affected	74.6 (15.7)	65.6 (19.4)	0.111^†^
Internal rotation unaffected	72.4 (22.5)	69.8 (17.9)	0.119^†^
**Daniels**			
**Affected side**			**0.008**^**μ**^
1	0 (0%)	0 (0%)	
2	1 (3.1%)	0 (0%)	
3	1 (3.1%)	0 (0%)	
4	10 (31.3%)	1 (3.1%)	
5	20 (62.5%)	31 (96.9%)	
Unaffected side			0.157^**μ**^
1	0 (0%)	0 (0%)	
2	0 (0%)	0 (0%)	
3	1 (3.1%)	0 (0%)	
4	4 (12.5%)	0 (0%)	
5	27 (84.4%)	32 (100%)	
**SPADI**			
Pain	30.1 (33.9)	6.1 (18.1)	**0.001**^†^
Disability	17.1 (19.9)	2.7 (7.9)	**0.001**^†^
Total	0.3 (0.3)	0.1 (0.2)	**0.001**^†^
**EAT-10**	16.7(10.7)	0 (0)	**0.001**^†^
**VAS**	2.1 (2.9)	0.03 (0.18)	**0.001**^**‡**^
**Location of swallowing disturbance**			**0.001**^**μ**^
None	4 (12.5)	31 (96.9)	
Pre-oral	4 (12.5)	0 (0)	
Oral	8 (25)	1 (3.1)	
Pharynx	13 (40.6)	0 (0)	
Pre-oral, oral and pharynx	3 (9.4)	0 (0)	

^μ^: Chi^2^ test

^†^: Mann-Whitney U test

^‡^: independent T-test

### Temporomandibular dysfunction

FAI was scored as no dysfunction for 12 sHNC and 24 controls, as light dysfunction for 7 sHNC and 8 controls; whereas 6 sHNC scored moderate dysfunction and 7 sHNC scored severe dysfunction (Table 3).

### Cervical function

sHNC revealed a significantly smaller cervical extension and inclination to both the affected and unaffected sides compared with controls (*p* < 0.05), whereas there were no statistically significant differences for the cervical flexion (*p* = 0.561) neither for cervical rotation, affected (*p* = 0.077) and unaffected sides (*p* = 0.194). sHNC reached statistically significant lower times on the DCFET while performing than the control group (*p* < 0.001).

### Shoulder function

Flexion and abduction on the affected side of the shoulder was significantly lower in sHNC than controls (*p* < 0.05), but there were no statistically significant differences for shoulder flexion and abduction on the unaffected side, neither for the external and internal rotation to both sides (*p* > 0.05).

Daniels and Worthingham’s muscle testing scale was scored below 5 for 12 sHNC whereas 1 control subject scored 4. Maximum score was reached for 20 sHNC and 31 controls. There was statistically significant difference between groups on the affected side (*p* = 0.008).

SPADI scores for pain, disability as well as the total score differed significantly between both groups (*p* < 0.001). Mean scores for sHNC were 30.1, 17.1 and 0.3 for the pain, disability and total subscales respectively, whereas 6.1, 2.7 and 0.04 were the scores reached on these subscales for the healthy controls (see Table 3).

### Swallowing function

Patients reached a mean of 16.7 on the EAT-10 questionnaire, which showed a statistically significant difference (*p* < 0.001) with the control group. The mean values from the VAS for the difficulty when swallowing were also statistically significant different between both groups (*p* < 0.001). Swallowing difficulty differences were statistically significant (*p* < 0.001) between sHNC and healthy controls. These swallowing difficulties were most reported by sHNC at the pharynx (40.6%), followed by the oral cavity (25%) and pre-oral (12.5%). Three patients (9.4%) reported disturbance at the 3 regions (pre-oral, oral and pharynx).

### Correlation between motor function and swallowing impairments

**Fig 1:**
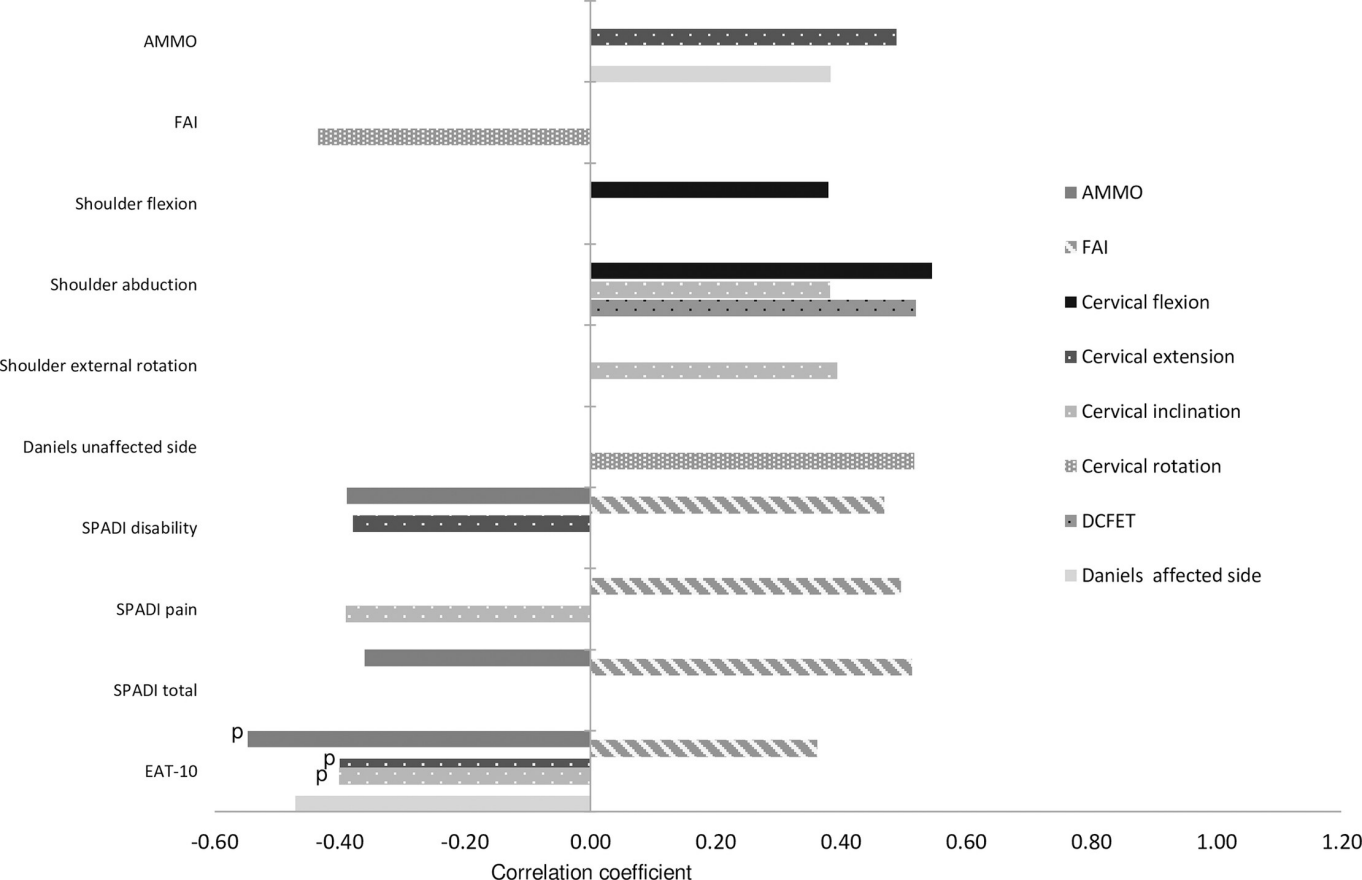
Significant correlations in sHNC between maximum mouth opening (MMO), temporomandibular dysfunction (TMD), cervical and shoulder function, and swallowing function (p<0.05). Pearson coefficient are indicated with a p. Spearman coefficient are all those that do not present a sign. DCFET: deep cervical flexor endurance test; EAT-10: Eating Assessment Tool; FAI: Fonseca Anamnestic Index; D&W: Daniels and Worthingham's muscle test score.

Table 3 shows the results for the measured variables. Fig 1 highlights the significant correlations (direct and inverse) detected. MMO correlated directly with the Daniels and Worthingham’s muscle test score on the affected side (p = 0.030), and inversely with the total SPADI and SPADI disability scores (p<0.05). The FAI correlated inversely with cervical rotation (p = 0.016) and directly with both SPADI subscales, the total SPADI score, and the EAT-10 score (p<0.05). Cervical flexion correlated directly with shoulder flexion and shoulder abduction (p<0.05). Cervical inclination correlated directly with shoulder abduction and shoulder external rotation (p<0.05), whereas cervical rotation showed a direct correlation with the Daniels and Worthingham’s muscle test score on the unaffected side (p = 0.003). The DCFET correlated directly with shoulder abduction (p = 0.003). The SPADI disability and pain indices correlated inversely with cervical extension and cervical inclination respectively (p<0.05). Finally, the EAT-10 score correlated inversely with MMO (p<0.001), cervical inclination (p = 0.002), cervical extension (p = 0.002) and the Daniels and Worthingham’s muscle test score on the affected side (p = 0.007), and directly with TMD (measured as FAI) (p = 0.045). No other correlations were found.

## Discussion

The aims of the current study were to study the correlation between MMO, TMD, motor function related to the cervical and shoulder regions and swallowing function in sHNC and to describe the differences on AMMO, TMD, cervical and shoulder functioning and swallowing function between sHNC and healthy matched controls.

Our results showed that MMO, TMD, cervical and shoulder motor function, and swallowing are considerably affected in sHNC compared to healthy controls. Moreover, correlations between MMO, TMD, cervical and shoulder function and swallowing function were found in sHNC.

### Survivors of head and neck cancer compared to healthy controls

MMO was found to be lower in patients compared to healthy controls. Trismus (restricted mouth opening) is a common complaint after treatment for HNC [31], appearing in about a quarter of all [17]. Indeed, mouth opening decreases by some 20% after treatment, especially with RT [13]—the consequence of damage and tissue fibrosis [32], possibly induced by apoptosis in response to radical‐mediated DNA damage [33].

The lower mean values of the sHNC compared to the control group for the cervical and shoulder AROM variables obtained in the present work agree with reductions consistently reported for populations with HNC (including sHNC) [5,16,18]. Extension and inclination to both sides on the cervical region were lower in sHNC. Flexion and abduction of the shoulder on the affected side were significantly lower in sHNC. Indeed, prospective cohort studies have indicated reduced AROM for both the cervical and shoulder areas at one year [34] and five years after treatment [35]. This may be the result of surgical stretching, compression or tissue burning [5]. Extensive surgery, such as RND, may reduce shoulder function via the sacrifice of the spinal accessory nerve [36]. In addition, the fibrosis that appears in radiated regions can reduce the AROM [35]. The loss of strength evidenced on this study in sHNC compared to healthy controls is also related to curative treatment: radiated-induced fibrosis reduces normal mobility of the regions affected; this may explain the decrease in the deep cervical flexor musculature. After resection or skeletonization of the accessory nerve, resection of the fascia surrounding the muscle or its devascularization, upper trapezius fibers may be no longer functional [16].

The decreased AROM added to the loss of strength may lead to a subjective perception of pain and disability during daily life activities, as shown in this study with the SPADI questionnaire. These results are in concordance with the most common shoulder impairments previously reported after ND [37]: pain, shoulder drop and loss of AROM.

Swallowing function was decreased in sHNC compared to healthy controls. This finding is in accordance with previous studies that evidenced swallowing impairments in patients treated for head and neck cancer [38,39]. RT causes harm to muscles involved in swallowing, leads inflammatory responses that induces fibrosis in time, atrophy, sensory loss, and thus, may result in dysphagia [14]. In this study, the most common reported location for swallowing disturbance was the pharynx, but this may be due to the clinical characteristics of our sample, as 35% of the sHNC participating on this study presented their tumor at hypopharynx or larynx levels.

### Correlation between motor and swallowing functions

Greater mobility and strength in the cervical region were found to be associated with the same in the shoulder. The cervical and shoulder regions are strongly connected via the origins and insertions of different muscles and by nerve branches involving the brachial plexus [40,41]. Moreover, previous research had stated that a surgical procedure in the cervical region involving cervical nerve roots might result in upper-extremity motor dysfunction [42].

The TMD (FAI) and SPADI results also correlated directly with each other; the greater the perception of TMD, the greater the shoulder pain and disability perceived. Although there may be less biomechanical interaction between the temporomandibular and shoulder regions than between the cervical and shoulder regions, this result suggests that, in sHNC, a relationship exists between loss of function in the former pair and the perception of pain and disability.

The correlation between MMO and cervical function shows the importance of an optimal cervical AROM in adequate mouth opening (and perhaps vice versa). A reduced MMO in sHNC is common due to radiation-induced fibrosis of the masticatory and cervical muscles [14].

It is known that the severity of TMD is related to the severity of cervical region disorders [43], although this has not been studied specifically in sHNC. An earlier prospective study reported the presence of TMD in patients diagnosed with HNC as possibly due to bruxism brought on by the anxiety and fear associated with a cancer diagnosis [44,45].

This is the first study to show an association between swallowing function and cervical AROM and shoulder strength in sHNC; a poorer cervical AROM and reduced shoulder strength are related to an increased perception of swallowing impairment. A previous cross-sectional study [46] reported a reduction in shoulder AROM in dependent older adults to be associated with dysphagia. No clear information exists regarding the relationship between swallowing and shoulder muscle function, but it may be that a loss of strength in the shoulder and cervical regions affects the position of the larynx and consequently its movement during swallowing [46]. One cross-sectional study suggests that patients with head and neck cancer are at risk of reduced physical functioning due to their undertaking lower levels of physical activity [47]; when physical effort is also reduced due to treatment, swallowing difficulties might be intensified.

### Strengths and limitations

A strength of this study is its use of objective and subjective methods of assessment; this allowed objective data (e.g., MMO, AROM, DCFET results) to be correlated with subjects’ perception of their difficulties. The small sample size, however, did not allow for regression analyses to check for differences due to tumor location, tumor size or the curative treatment received. Finally, the present sHNC had completed their treatment in the previous 6 to 36 months. In future work with larger samples it might be advisable to stratify patients in terms of the time elapsed since treatment ended and include the association of oncology treatment parameters with different levels of disability.

### Clinical implications

The present results suggest that physiotherapy to improve the MMO and cervical and shoulder AROM and strength in sHNC may help reduce swallowing difficulties. The associations detected between different body regions indicate treatment strategies may need to involve the face, cervical and shoulder regions. Mouth opening is mainly treated via the use of jaw-mobilizing devices and exercises once any radiotherapy is completed. The cervical region may be treated with stretching exercises and cervical traction (horizontal plane), while the shoulder should be subjected to passive and active range of motion exercises to prevent adhesive capsulitis [48]. Both regions should be treated as soon as possible after any surgery.

### Future research

Future studies should explore the correlations between the measured variables in larger samples of sHNC with different clinical characteristics. The results obtained may allow for clinical trials of specific treatments aimed at reducing swallowing difficulty.

## Conclusion

sHNC present lower MMO, higher perception of TMD, lower cervical and shoulder function which are inter-related to each other’s, besides greater swallowing impairments compared to healthy controls. The degree of swallowing impairment perceived by sHNC is associated with a lower MMO, higher perception of TMD, poorer cervical AROM (specifically cervical extension and inclination) and reduced shoulder strength. These impairments may be induced by the surgical procedure and the side effects of RT and chemotherapy. Physiotherapy might help improve these variables, reducing the perception of swallowing difficulty.
